# Subthalamic segmentations in relation to deep brain stimulation volumes in Parkinson’s disease

**DOI:** 10.1007/s00701-026-06930-3

**Published:** 2026-06-01

**Authors:** Alexander Calvano, Yiming Xiao, Kenan Steidel, Philipp A. Loehrer, Marina C. Ruppert-Junck, Christopher Nimsky, Lars Timmermann, Miriam H. A. Bopp, David J. Pedrosa

**Affiliations:** 1 Department of Neurology, Philipps-University Marburg, Baldingerstraße, 35043 Marburg, Germany; 2 Department of Computer Science and Software Engineering, Concordia University, Montreal, Québec Canada; 3 Donders Institute for Brain, Cognition and Behaviour, Centre of Expertise for Parkinson & Movement Disorders, Radboud University Medical Centre, 6525 EN Nijmegen, The Netherlands; 4 Centre of Mind, Brain and Behaviour, Philipps-University Marburg, Marburg, Germany; 5 Department of Neurosurgery, Philipps-University Marburg, Marburg, Germany

**Keywords:** Parkinson’s disease, Movement disorders, Subthalamic nucleus, Deep brain stimulation, Label fusion

## Abstract

**Purpose:**

Accurate segmentation of the subthalamic nucleus (STN) is paramount for optimising outcomes under deep brain stimulation (DBS) in Parkinson’s disease (PD). Clinically available tools like Brainlab Elements (BL-E) enable automated segmentations for surgical planning, yet their spatial relationship with postoperative volumes of tissue activated (VTAs) remains insufficiently characterised. Using multi-atlas segmentation (MAS) as an external anatomical reference, we compared the spatial correspondence of STN segmentations derived from BL-E with effective VTAs following monopolar contact review.

**Methods:**

We analysed imaging data from 40 PD patients with chronic STN-DBS. Segmentations were obtained using BL-E based on T1w and T2w scans and MAS derived from a library of 20 manually segmented midbrain nuclei atlases. Spatial correspondence was assessed using Dice Coefficients, Jaccard Indices, and Euclidean centroid distances. Distances between VTA centroids and clinically established settings for STN-DBS were calculated to evaluate targeting consistency. Statistical differences between metrics were assessed using Wilcoxon signed-rank tests.

**Results:**

BL-E segmentations demonstrated superior spatial correspondence with VTAs compared to MAS, with smaller Euclidean distances between centroids (*p* < 0.001). Dice Coefficients and Jaccard Indices showed no significant differences (*p* = 0.18). VTA centroid distances to the most efficient stimulation location were consistent across hemispheres (left: 2.54 mm [1.92–3.25]; right: 2.87 mm [1.85–3.82]) MAS targets were positioned more inferiorly and anteriorly compared to BL-E targets.

**Conclusion:**

Clinically applied VTAs showed good spatial correspondence with planning segmentations, suggesting within-workflow reproducibility but not superior correspondence to anatomical ground truth per se. Future studies should incorporate connectomic information to more accurately reflect the functional relevance of stimulation and its therapeutic effects.

**Supplementary Information:**

The online version contains supplementary material available at 10.1007/s00701-026-06930-3.

## Introduction

Accurate preoperative planning and lead placement are indispensable for the clinical benefit of deep brain stimulation (DBS) in Parkinson’s disease (PD). Tools like Brainlab Elements (BL-E) utilise a patented synthetic tissue model for patient-specific anatomical segmentation of targets such as the subthalamic nucleus (STN), integrating multiple magnetic resonance imaging (MRI) tissue classes through modality-specific spatial registrations [11, 31]. The BL-E segmentation approach is designed for clinical workflows, with close agreement between segmented STN boundaries and electrophysiological measurements [40]. Since these delineations directly guide surgical targeting, the resulting stimulation volume is inherently shaped by the anatomical model used during planning. Assessing the spatial concordance between segmentation and clinically applied stimulation fields is therefore important to evaluate within-workflow reproducibility. To date, however, the relationship between automated STN segmentations and postoperative volumes of tissue activated (VTAs) has not been systematically characterised.

Multi-atlas segmentation (MAS) provides a research-oriented alternative by combining multiple atlases registered to a target image [27]. Majority voting resolves conflicts among registered atlases [4, 48]. MAS demonstrates accurate segmentation performance [2, 20] by leveraging multiple registrations to capture higher inter-subject anatomical variability while reducing the impact of occasional registration errors [42]. In addition, the fusion of multiple atlases helps mitigate segmentation inconsistencies between templates and reduces label interpolation inaccuracies [27, 41]. MAS may therefore serve as an external anatomical reference to evaluate the spatial alignment of clinically implemented targeting strategies.

In this study, we assessed the within-workflow reproducibility of the BL-E software by quantifying the spatial correspondence between BL-E- and MAS-derived STN segmentations and clinically applied VTAs informed by monopolar review.

## Methods and materials

### Patients

Data from 40 patients with PD (16 females, 58.2 ± 7.2 years) from the Department of Neurology at the Philipps-University Marburg were retrospectively evaluated, who had undergone chronic STN-DBS electrode surgery (Boston Scientific Neuromodulation Corporation, Valencia, CA 91355, USA). The study was approved by the local ethics committee (reference 22/29) and was conducted adhering to the principles of the latest version of the Declaration of Helsinki. Tables containing descriptive analyses and individual stimulation settings are included as Online Resources.

### Clinical assessment

A standardised monopolar contact review was conducted for each patient three months after surgery [47]. To this end, each of the 16 electrode contacts was tested in monopolar configuration, with stimulation amplitude increased in 0.5 mA steps up while pulse width and frequency were held constant at 60 µs and 130 Hz, respectively. Motor performance was assessed at each current intensity, and testing continued until either intolerable side effects occurred or a maximum amplitude of 4 mA was reached. No image-guided refinement of stimulation parameters was performed. Global motor severity was evaluated using the motor scale of the MDS-sponsored revised version of the Unified Parkinson’s Disease Rating Scale (MDS-UPDRS-III) [19]. Percentage improvement between stimulation OFF and ON conditions was calculated as (1):

1 $$Improvement (\%)=\frac{MDS-UPDR{S-III}_{OFF}-MDS-UPDR{S-III}_{ON}}{MDS-UPDR{S-III}_{OFF}}\times 100$$

### VTA modelling

The combination providing the optimal therapeutic response was selected for VTA modelling according to the Lead-DBS pipeline (version 2.6) [23]. Briefly, after coregistration of pre- and postoperative scans, all images were spatially normalised into the standard MNI152 space [18]. Leads were localised with the PaCER algorithm, including their orientation [26]. Using a finite element model, stimulation volumes were computed by estimating the gradient distribution of the electrical charge in space on a tetrahedral mesh comprising grey matter, white matter, electrode contacts, and insulation [24]. A threshold of 0.2 V/mm was applied to define the electric field extent [8, 9]. For each patient, bilateral VTAs were resampled to match space dimensions of the reference image using *antsApplyTransforms*, as implemented in Advanced Normalization Tools (ANTs) [5].

### Image processing

Pre-operative MRI was acquired with a 3 T MRI system (Tim Trio, Siemens Healthineers, Erlangen, Germany) [10]. Image processing was performed at an isotropic resolution of 1 × 1 × 1 mm^3^. The data for pre-operative T1w and the T2w scans were pre-processed with denoising [33], N4 field inhomogeneity correction [44], and skull stripping with BEaST [16]. The T1w and T2w data were coregistered with a rigid body transform and linearly registered with a 12-parameter affine transform to the ICBM152-2009c template space [18]. After pre-processing, an automatic multi-atlas label fusion technique was employed to segment the STN from each patient’s T2w data [49]. A library of 20 manually segmented midbrain nuclei atlases [49] was independently non-linearly registered to each patient’s T2w MRI in MNI152 space using ANTs, and labels were transferred to the patient’s data through the non-linear transform. The final segmentation was determined by a majority vote scheme, where the label with the most votes from the atlases was retained.

STN segmentations were also obtained with the Elements Segmentation Basal Ganglia module (Release 4.0; Brainlab AG, Munich, Germany) as part of BL-E, integrating T2w scans for optimal contrast of basal ganglia structures and T1w images for anatomical reference and registration. The resulting segmentation masks were post-processed using tools from the FMRIB Software Library (FSL) 6.0.5.2 (https://fsl.fmrib.ox.ac.uk/fsl). Masks were first reoriented in native space via FSL’s *fslreorient2std* tool [28]. A linear rigid-body transformation was then applied using an identity transformation matrix with nearest-neighbour interpolation to preserve label integrity. Finally, each segmentation mask was linearly transformed to standard MNI152 space through the previously obtained affine matrices [29].

### Statistical analysis

The correspondence between each STN segmentation and effective VTAs was assessed using established spatial similarity metrics. The Dice Coefficient and Jaccard Index quantified volumetric overlap between binary masks and the proportion of shared volume relative to their union, respectively. Moreover, Euclidean distances between centroids of each STN vs. VTA pair were calculated. To evaluate targeting consistency across hemispheres, we measured distances between VTA centroids and the most efficient stimulation location in the STN for PD (left: x = −12.68 mm, y = −13.53 mm, z = −5.38 mm; right: x = 12.50 mm, y = −12.72 mm, z = −5.38 mm) [14]. Spearman correlation was performed to assess the relationship between spatial metrics and motor improvement. Scripts for automatisation and visualisation were written in Bash and Python using the packages nibabel, numpy, pandas, and seaborn. Statistical analyses were performed in R (version 4.3.1) [43]. Wilcoxon signed-rank tests were applied to compare spatial metrics across segmentations, with results expressed as median and interquartile ranges (Q1–Q3). Statistical significance was defined as *p* < 0.05.

## Results

**Fig. 1:**
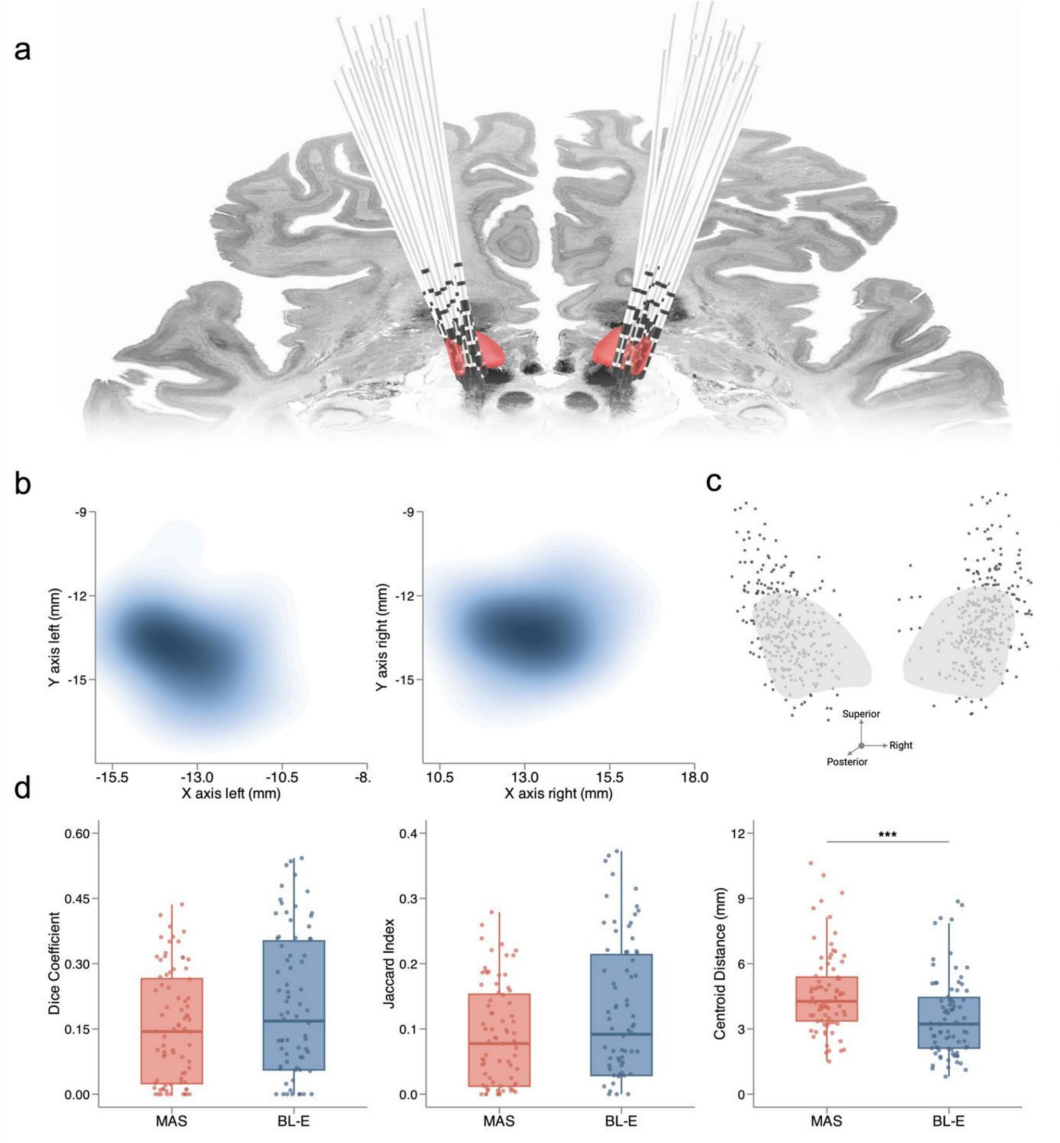
DBS lead locations and analysis of spatial metrics. **a** Electrode positions in MNI152 space. The subthalamic nucleus (STN) is illustrated in red; parcellation according to the DISTAL minimal atlas [17]. **b** Heatmap of volumes of tissue activated (VTA) centroids in MNI152 space for the left and right hemisphere. Axes denote left-to-right (x) and posterior-to-anterior (y) coordinates. **c** Spatial distribution of all electrode contacts projected onto the left and right STN. **d** Comparison of spatial correspondence metrics between multi-atlas segmentation (MAS, red) and Brainlab Elements (BL-E, blue). ***p < 0.001 (Wilcoxon signed-rank test)

The location of all DBS leads in MNI152 space is shown in Fig. 1A. The average coordinates of VTA centroids on the left and right were: x = −13.5 mm, y = −13.8 mm, and z = −5.9 mm; x = 13.2 mm, y = −13.0 mm, and z = −6.2 mm, respectively (Fig. 1B). The distances between VTA centroids and the most efficient stimulation localisation in the STN were 2.54 mm [1.92–3.25] for the left hemisphere and 2.87 mm [1.85–3.82] for the right hemisphere, indicating consistent targeting for both sides. VTA volumes did not differ significantly between hemispheres (left: 55.50 mm^3^ [38.75–93.25]; right: 67.0 mm^3^ [50.25–88.50]; *p* = 0.74). Dice Coefficients were lower for MAS (0.14 [0.02–0.27]) than for BL-E (0.17 [0.06–0.35]), although this difference did not attain statistical significance (*p* = 0.18). A similar relationship was observed for the Jaccard indices (MAS: 0.08 [0.01–0.15]; BL-E: 0.09 [0.03–0.21]; *p* = 0.18). The Euclidean distances between VTAs and STN centroids was significantly greater for MAS (4.27 [3.37–5.38]) compared to BL-E segmentations (3.22 [2.12–4.45]; *p* < 0.001) (Fig. 1D). Analysis of centroid locations revealed that MAS targets were positioned more inferiorly (*p* < 0.001) and anteriorly (*p* < 0.001) compared to BL-E targets (Table 1). No associations were observed between motor improvement and spatial metrics (Dice coefficient: rho = –0.04, *p* = 0.80; Jaccard index: rho = –0.04, *p* = 0.80; Centroid distance: rho = 0.04, *p* = 0.83).

**Table 1:** Comparison of spatial metrics, segmentation volumes, and centroid locations in MNI152 space

	MAS						BL-E						***p*** value
Dice	0.14 [0.02–0.27]						0.17 [0.06–0.35]						0.18
Jaccard	0.08 [0.01–0.15]						0.09 [0.03–0.21]						0.18
Centroid (mm)	4.27 [3.37–5.38]						3.22 [2.12–4.45]						< **0.001**
Size (mm^3^)	119.50 [109.80–130.0]						131.50 [123.0–142.0]						< **0.001**
	Side						Side						
	Right			Left			Right			Left			
	X	Y	Z	X	Y	Z	X	Y	Z	X	Y	Z	
MNI centroids	10.74	−11.85	−8.43	−10.61	−11.77	−8.41	12.52	−12.43	−6.50	−12.25	−12.76	−6.54	

Results are reported as median and interquartile range. *MAS* multi-atlas segmentation, *BL-E* Brainlab Elements. Significant results (*p* < 0.05) are shown in bold

## Discussion

In this study, we assessed the spatial correspondence of an automated segmentation approach for the subthalamic nucleus used in surgical planning with clinically applied volumes of tissue activated. Multi-atlas segmentation was used as an external reference. Delineations generated by the commercially available Brainlab Elements software exhibited significantly lower centroid distances to VTAs compared to those obtained from MAS, suggesting within-workflow reproducibility. Notably, overlap metrics such as the Dice coefficient and Jaccard index did not show significant differences between the two segmentation methods.

Automatic segmentation of midbrain structures has become increasingly important for surgical planning and postoperative imaging analysis following DBS [34]. In our study, BL-E segmentations were used for preoperative planning and thus inherently influenced the anatomical location of the resulting VTAs. While MAS served as an external reference to contextualise spatial concordance, it does not eliminate the potential influence of within-workflow circularity. Consequently, our findings primarily demonstrate consistency within the BL-E pipeline rather than superior correspondence to anatomical ground truth.

BL-E and MAS rely on distinct modelling strategies: BL-E performs segmentation by generating modality-specific atlases tailored to the contrast characteristics of each MRI modality. This process includes applying multiple independent nonlinear registrations and using structure-specific weighting to delineate the target in a patient-specific manner [11, 40]. In contrast, MAS registers multiple labeled atlases to the patient’s image and fuses the resulting labels into a consensus segmentation [49]. The inferior and anterior displacement observed for MAS relative to BL-E suggests that segmentation strategy meaningfully influences the geometric representation of the STN in standard space. Such differences may be relevant for multi-centre studies that aggregate datasets processed with heterogeneous segmentation pipelines. One possible explanation for displacement is that the MAS atlas library may not fully capture the anatomical variability present in the PD population [3, 49]. Given that the morphology of subcortical structures varies with ageing, disease progression [30], and across parkinsonian subtypes [32], atlas composition and population matching likely affect spatial precision.

Spatial metrics are frequently employed in neuroimaging research to evaluate segmentation accuracy and quantify spatial relationships between anatomical structures [31, 37, 40]. In our study, however, spatial metrics can only provide a rough estimate of structure-function relationships. This consideration arises partly because stimulation is typically delivered to the dorsolateral region of the STN rather than its geometric centre [14]. As such, centroid distances may not adequately capture the spatial features mediating clinical effects. Associations between overlap metrics and clinical benefit are therefore usually assessed at the hemispheric and subregional levels to account for asymmetry in parkinsonian motor symptoms [38] and the functional organisation of the STN [21]. In our dataset, however, both segmentation approaches lacked such subparcellation, restricting our ability to examine the relationship between VTAs and the motor subregion specifically. These considerations likely reduced sensitivity and may explain the absence of detectable associations between spatial metrics and clinical improvement.

While centroid distances and overlap metrics allowed us to interpret the spatial relationship between stimulation volumes and anatomical segmentations, they remain an imperfect surrogate for clinical effectiveness. Growing evidence suggests that optimal motor control is achieved by modulating connected brain circuits rather than by improving spatial concordance between stimulation volumes and a single anatomical structure [25]. Given its widespread cortical projections, the STN is uniquely positioned to facilitate therapeutic neuromodulation across several subcircuits within distinct symptom domains [22, 39]. At the subcortical level, symptom-specific fibre tracts exhibit a rostrocaudal gradient, with tremor-related pathways appearing to be positioned more posteriorly. Tremor improvement has primarily been linked to connections involving the primary motor cortex, whereas hypokinetic symptoms are associated with a stronger premotor distribution [39]. In line with this network perspective, stimulation of hyperdirect and cerebellothalamic pathways near the caudal Zona incerta effectively suppresses motor symptoms and reduces internal capsule side effects compared to direct STN targeting [6, 7, 36]. Similarly, targeting of white matter fibres such as the dentatorubrothalamic tract has been proposed as a marker for imaging-guided contact selection in tremor control [45], further supporting the concept of circuit-level therapeutic effects of DBS. The growing recognition of connectivity profiles as key determinants of clinical outcome may help explain the absence of differences in overlap metrics between BL-E and MAS. Accordingly, future studies may benefit from incorporating connectomic data in order to more accurately reflect the relationship between stimulation and therapeutic effects [9].

Several limitations warrant consideration. For group-level analysis, spatial metrics were computed in MNI 152 standard space, which may have introduced residual misalignment from non-linear transformations. This issue is particularly pertinent for small subcortical structures such as the STN, where minor inaccuracies can meaningfully influence centroid localisation, overlap estimates, and mapping of DBS response [35]. Despite recent advances in normalisation algorithms [46], systematic spatial misregistration may account for a considerable proportion of variability in lead localisation [1]. In addition, low contrast-to-noise ratio [35] and inter-individual variability in STN morphology and localisation [15, 30] may further compromise anatomical fidelity in standard-space analyses. Partial volume effects at the employed voxel resolution of 1 × 1 × 1 mm^3^ cannot be fully eliminated, and given the well-known limitations of STN delineation at field strengths below 7 T [12, 13], boundary voxels may contain mixed signal from adjacent structures, including the zona incerta, the substantia nigra, and surrounding white matter [13]. Furthermore, stimulation settings were derived from standardised monopolar reviews three months after surgery and may not reflect optimised chronic stimulation settings. Finally, the single-centre design and the relatively small sample size may hamper the generalisability of our findings.

In conclusion, our findings indicate that BL-E segmentations show lower centroid distances to clinically applied VTAs compared to MAS, suggesting within-workflow reproducibility for the automated segmentation software. Nevertheless, they do not imply superior correspondence to anatomical ground truth per se. Future studies should incorporate connectomic information to more accurately reflect the functional relevance of stimulation and its therapeutic effects.

## Supplementary Information

Below is the link to the electronic supplementary material.

ESM 1 Supplementary Material 1 (DOCX 26.6 KB)

## Data Availability

The code used for the analyses in this report is available in an online repository (https:/github.com/AlexanderCalvano/retroDBS.git). The data that support the findings of this study are not publicly available due to privacy or ethical restrictions but are available from the corresponding author (AC) upon request.
