# Supplementary material for: Subthalamic segmentations in relation to deep brain stimulation volumes in Parkinson’s disease
Source: Acta Neurochir (Wien). 2026 Jun 1;168(1):125. doi: 10.1007/s00701-026-06930-3 (PMC13230244; doi:10.1007/s00701-026-06930-3)
Supplement: Supplementary file 1 — Supplementary Material 1 (DOCX 26.6 KB) [file 701_2026_6930_MOESM1_ESM.docx]

**Supplementary Tables**

| **ID** | **Age** [years] | **MDS-UDPRS III** (Med ON, Stim ON) | **Subtype** | **LEDD** [mg] | **Side prevalence** |
| --- | --- | --- | --- | --- | --- |
| 1 | 42 | 13 | equivalent‑type | 475 | left |
| 2 | 64 | 15 | tremor-dominant | 532 | right |
| 3 | 53 | 18 | hypokinetic-rigid | 1160 | left |
| 4 | 48 | 19 | equivalent‑type | 402 | right |
| 5 | 66 | 14 | hypokinetic-rigid | 1534 | right |
| 6 | 46 | 16 | equivalent‑type | 1716 | right |
| 7 | 57 | 23 | equivalent‑type | 575 | left |
| 8 | 64 | 7 | hypokinetic-rigid | 575 | left |
| 9 | 62 | 5 | equivalent‑type | 1150 | left |
| 10 | 54 | 69 | hypokinetic-rigid | 1365 | left |
| 11 | 61 | 18 | hypokinetic-rigid | 566 | left |
| 12 | 60 | 38 | tremor-dominant | 560 | left |
| 13 | 48 | 11 | hypokinetic-rigid | 773 | right |
| 14 | 58 | 20 | equivalent‑type | 575 | right |
| 15 | 61 | 18 | tremor-dominant | 752 | right |
| 16 | 38 | 10 | hypokinetic-rigid | 420 | left |
| 17 | 51 | 18 | equivalent‑type | 1275 | left |
| 18 | 52 | 33 | tremor-dominant | 3475 | left |
| 19 | 59 | 19 | hypokinetic-rigid | 1209 | left |
| 20 | 53 | 9 | hypokinetic-rigid | 510 | right |
| 21 | 65 | 21 | equivalent‑type | 610 | right |
| 22 | 70 | 32 | hypokinetic-rigid | 400 | right |
| 23 | 60 | 16 | equivalent‑type | 900 | right |
| 24 | 63 | 26 | equivalent‑type | 746 | right |
| 25 | 60 | 28 | equivalent‑type | 52 | left |
| 26 | 59 | 44 | tremor-dominant | 405 | right |
| 27 | 52 | 8 | ttremor-dominant | 507 | left |
| 28 | 51 | 33 | tremor-dominant | 884 | left |
| 29 | 55 | 30 | hypokinetic-rigid | 650 | right |
| 30 | 70 | 59 | tremor-dominant | 400 | right |
| 31 | 59 | 12 | tremor-dominant | 80 | right |
| 32 | 63 | 5 | equivalent‑type | 415 | left |
| 33 | 70 | 12 | hypokinetic-rigid | 252 | left |
| 34 | 63 | 7 | hypokinetic-rigid | 845 | left |
| 35 | 58 | 17 | hypokinetic-rigid | 460 | right |
| 36 | 67 | 33 | equivalent‑type | 452 | right |
| 37 | 61 | 29 | tremor-dominant | 410 | right |
| 38 | 61 | 12 | hypokinetic-rigid | 200 | right |
| 39 | 54 | 25 | equivalent‑type | 215 | left |
| 40 | 68 | 23 | tremor-dominant | 475 | right |
| **Mean ± SD** | 58.2 ± 7.5 | 21.6 ± 13.7 | NA | 473.9 ± 587.6 | NA |

**Supplementary Table 1: Clinical details.** Abbreviations: DD: Disease duration, LEDD: Levodopa equivalent daily dose, MDS-UPDRS: Movement Disorder Society-Unified Parkinson’s Disease Rating Scale, NA: Not applicable, SD: Standard deviation

| **ID** | **left STN** | | **right STN** | |
| --- | --- | --- | --- | --- |
|  | active contacts | amplitude [mA] | active contacts | amplitude [mA] |
| 1 | 5-(29%), 6-(16%), 7-(13%), 8-(42%) | 2.2 | 13-(27%), 14-(11%), 15-(7%), 16-(55%) | 2.2 |
| 2 | 3-(50%), 4-(50%) | 2.3 | 10-(38%), 12-(12%), 13-(38%), 15-(12%) | 2.4 |
| 3 | 2-(100%) | 1.4 | 12-(100%) | 1.5 |
| 4 | 6-(15%), 7-(45%), 8-(40%) | 3.4 | 13-(20%), 14-(20%), 16-(60%) | 3.8 |
| 5 | 5-(7%), 6-(44%), 7-(19%), 8-(30%) | 1.4 | 10-(18%), 11-(16%), 12-(16%), 13-(18%), 14-(16%), 15-(16%) | 1.4 |
| 6 | 2-(34%), 3-(33%), 4-(33%) | 2.4 | 13-(50%), 14-(50%) | 2.4 |
| 7 | 2-(100%) | 1.8 | 13-(34%), 14-(33%), 15-(33%) | 2 |
| 8 | 2-(34%), 3-(33%), 4-(33%) | 4 | 10-(5%), 11-(15%), 13-(60%), 15-(20%) | 5.7 |
| 9 | 2-(34%), 3-(33%), 4-(33%) | 2.5 | 10-(34%), 11-(33%), 12-(33%) | 2.4 |
| 10 | 5-(20%), 6-(20%), 7-(40%), 8-(20%) | 2.3 | 13-(10%), 14-(25%), 15-(40%), 16-(25%) | 3 |
| 11 | 3-(10%), 4-(10%), 6-(40%), 7-(40%) | 2 | 10-(34%), 11-(33%), 12-(33%) | 1.5 |
| 12 | 2-(34%), 3-(33%), 4-(33%) | 1.8 | 10-(34%), 11-(33%), 12-(33%) | 2.4 |
| 13 | 2-(14%), 3-(13%), 4-(13%), 5-(20%), 6-(20%), 7-(20%) | 1.6 | 10-(20%), 11-(20%), 12-(20%), 13-(13%), 14-(13%), 15-(14%) | 2.6 |
| 14 | 5-(30%), 6-(30%), 7-(30%), 8-(10%) | 3 | 10-(15%), 11-(15%), 12-(45%), 13-(10%), 15-(30%) | 3 |
| 15 | 2-(30%), 3-(30%), 5-(30%) | 2.8 | 13-(31%), 14-(30%), 15-(30%), 16-(9%) | 3.0 |
| 16 | 2-(10%), 3-(10%), 4-(30%), 5-(10%), 6-(10%), 7-(30%) | 1.3 | 10-(10%), 11-(20%), 12-(30%), 13-(20%), 14-(10%), 15-(10%) | 1.9 |
| 17 | 1-(90%), 4-(10%) | 0.9 | 9-(100%) | 0.3 |
| 18 | 2-(10%), 4-(30%), 5-(15%), 7-(45%) | 4.2 | 13-(34%), 14-(33%), 15-(33%) | 3.6 |
| 19 | 2-(34%), 3-(33%), 4-(33%) | 2.2 | 10-(50%), 11-(50%) | 2 |
| 20 | 2-(34%), 3-(33%), 4-(33%) | 1.7 | 10-(34%), 11-(33%), 12-(33%) | 1.6 |
| 21 | 2-(60%), 5-(40%) | 2.2 | 10-(40%), 12-(40%), 13-(10%), 15-(10%) | 1.9 |
| 22 | 4-(30%), 7-(50%), 8-(20%) | 2.4 | 11-(50%), 12-(25%), 13-(25%) | 1.5 |
| 23 | 2-(5%), 3-(3%), 4-(12%), 5-(22%), 6-(11%), 7-(47%) | 2 | 9-(10%), 10-(6%), 11-(24%), 12-(60%) | 2 |
| 24 | 2-(2%), 3-(33%), 4-(34%), 5-(3%), 6-(14%), 7-(14%) | 4.6 | 10-(11%), 11-(25%), 12-(4%), 13-(16%), 14-(38%), 15-(6%) | 4.6 |
| 25 | 4-(50%), 7-(50%) | 2 | 10-(40%), 11-(40%), 12-(20%) | 4.3 |
| 26 | 2-(24%), 3-(23%), 4-(23%), 5-(10%), 6-(10%), 7-(10%) | 1.6 | 10-(24%), 11-(23%), 12-(23%), 13-(10%), 14-(10%), 15-(10%) | 1.5 |
| 27 | 4-(10%), 7-(30%), 8-(60%) | 1.2 | 10-(20%), 11-(40%), 13-(40%) | 3 |
| 28 | 3-(20%), 4-(20%), 5-(40%), 6-(20%) | 2.9 | 9-(5%), 11-(20%), 12-(20%), 13-(55%) | 1.2 |
| 29 | 6-(30%), 7-(30%), 8-(40%) | 1.7 | 13-(40%), 15-(40%), 16-(20%) | 1.5 |
| 30 | 2-(34%), 3-(33%), 4-(33%) | 0.5 | 10-(34%), 11-(33%), 12-(33%) | 0.8 |
| 31 | 2-(42%), 3-(25%), 4-(9%), 5-(19%), 6-(5%) | 2.6 | 11-(5%), 12-(50%), 13-(40%), 14-(5%) | 3.4 |
| 32 | 1-(20%), 2-(28%), 3-(26%), 4-(26%) | 0.5 | 13-(34%), 14-(33%), 15-(33%) | 1 |
| 33 | 2-(34%), 3-(33%), 4-(33%) | 0.8 | 10-(34%), 11-(33%), 12-(33%) | 0.8 |
| 34 | 5-(30%), 6-(30%), 7-(30%), 8-(10%) | 2.4 | 10-(8%), 11-(6%), 12-(6%), 13-(28%), 14-(26%), 15-(26%) | 2.5 |
| 35 | 2-(40%), 3-(40%), 4-(20%) | 2.2 | 9-(60%), 11-(15%), 14-(25%) | 1.5 |
| 36 | 2-(34%), 3-(33%), 4-(33%) | 2.5 | 10-(34%), 11-(33%), 12-(33%) | 2.1 |
| 37 | 2-(24%), 3-(23%), 4-(23%), 5-(10%) | 2.6 | 10-(24%), 11-(23%), 12-(23%), 13-(10%) | 2.5 |
| 38 | 5-(34%), 6-(33%), 7-(33%) | 1.2 | 13-(34%), 14-(33%), 15-(33%) | 0.9 |
| 39 | 1-(20%), 4-(80%) | 5.1 | 9-(20%), 10-(40%), 11-(25%), 12-(15%) | 2.8 |
| 40 | 5-(50%), 6-(50%) | 2.3 | 15-(80%), 16-(20%) | 2.3 |

**Supplementary Table 2: Individual DBS programs of all participants.** Pulse width was at 60 µs and frequency at 130 Hz in all patients.
